# The Sense of Coherence and Health Behavior of Men with Alcohol Addiction

**DOI:** 10.3390/ijerph19148650

**Published:** 2022-07-16

**Authors:** Mateusz Curyło, Marlena Rynkiewicz-Andryśkiewicz, Przemysław Andryśkiewicz, Marcin Mikos, Dariusz Lusina, Jan W. Raczkowski, Grzegorz Juszczyk, Artur Kotwas, Katarzyna Sygit, Kamila Kmieć, Elżbieta Cipora, Mateusz Kaczmarski, Tomasz Banaś, Łukasz Strzępek, Andrzej Śliwczyński, Jan Krakowiak, Jakub Świtalski, Olga Partyka, Aleksandra Czerw

**Affiliations:** 1 Department of Orthopaedic and Post-Traumatic Rehabilitation, Medical University of Lodz, 90-647 Lodz, Poland; mateusz.curylo@azmmedical.pl (M.C.); jan.raczkowski@umed.lodz.pl (J.W.R.); 2 Department of Treatment of Alcohol Abstinence Syndromes, Independent Public Healthcare Facility in Lezajsk, 37-300 Lezajsk, Poland; marlenarynkiewicz@gmail.com (M.R.-A.); p.andryskiewicz@gmail.com (P.A.); 3 Faculty of Medicine and Health Sciences, Andrzej Frycz Modrzewski Krakow University, 30-705 Krakow, Poland; mikos@ziz.com.pl; 4 Department of Orthopedics and Traumatology, University Hospital in Krakow, 30-688 Krakow, Poland; 8darek@gmail.com; 5 Department of Public Health, Medical University of Warsaw, 02-091 Warsaw, Poland; grzegorz.juszczyk@wum.edu.pl; 6 Subdepartment of Social Medicine and Public Health, Department of Social Medicine, Pomeranian Medical University in Szczecin, 70-204 Szczecin, Poland; artur.kotwas@pum.edu.pl; 7 Faculty of Health Sciences, Calisia University, 62-800 Kalisz, Poland; ksygit@poczta.onet.pl (K.S.); k.kmiec@akademia.kalisz.pl (K.K.); 8 Medical Institute, Jan Grodek State University in Sanok, 38-500 Sanok, Poland; ecipora@up-sanok.edu.pl (E.C.); mkaczmarski@up-sanok.edu.pl (M.K.); 9 Department of Gynecology and Obstetrics, Jagiellonian University Medical College, 31-501 Krakow, Poland; tbanas@mp.pl; 10 Department of Radiotherapy, Maria Sklodowska-Curie Institute-Oncology Centre, 31-115 Cracow, Poland; 11 Department of General Surgery, Regional Public Hospital in Bochnia, 32-700 Bochnia, Poland; strzepeklukasz@wp.pl; 12 Branch Didactic Center in Warsaw, University of Humanities and Ekonomics in Lodz, 90-212 Lodz, Poland; andrzej.sliwczynski.ahe@gmail.com; 13 Department of Social Medicine, Medical University of Lodz, 90-419 Lodz, Poland; jan.krakowiak@umed.lodz.pl; 14 Department of Health Economics and Medical Law, Medical University of Warsaw, 02-091 Warsaw, Poland; jswitalski@wum.edu.pl (J.Ś.); opartyka@pzh.gov.pl (O.P.); 15 Department of Economic and System Analyses, National Institute of Public Health NIH-National Research Institute, 00-791 Warsaw, Poland

**Keywords:** sense of coherence, alcoholism, pro-health behavior, salutogenesis

## Abstract

Introduction: Alcohol dependence is one of the world’s major health challenges. The salutogenic concept of health developed by Antonovsky focuses on the search for resources and factors supporting health. Its basic concept of the sense of coherence (SOC) focuses on strengthening the global orientation of the patient, and creating permanent internal resources that translate into the improvement of pro-health behavior, including the fight against alcoholism. Objective: The objective of this study was to determine the correlation between individual factors and the SOC as well as the influence of the SOC concept on pro-health behavior of people addicted to alcohol. Materials and methods: The study group consisted of 110 men undergoing treatment in an addiction treatment ward. To check the level of the SOC, two standardized questionnaires, Antonovsky’s “SOC-29 Life Orientation Questionnaire” and Juczyński’s “Health Behaviour Inventory”, were used. The correlation coefficient between the sociodemographic variables was checked using the Pearson’s r test. Results: A positive correlation was found with the intensity of pro-health behaviors for three sociodemographic variables. In people aged 43–65 (r = 0.299; *p* = 0.030), people with primary/vocational education (r = 0.276; *p* = 0.015), and respondents living in rural areas (r = 0.303; *p* = 0.028) a greater SOC was associated with pro-health behaviors. Conclusions: Individuals addicted to alcohol are characterized by a low SOC and a low level of pro-health behaviors. Strengthening the internal level of the SOC can constitute an element of addiction therapy when introducing health education to prepare the patient for independent life in sobriety.

## 1. Introduction

According to research, alcohol is the most commonly consumed psychoactive substance in the world, influencing the functioning of the brain and the behavior of an individual. Over the years, it has been identified as a risk factor in over 200 diagnoses [1]. WHO estimates that 5.3% of all deaths in the world annually are caused by excessive alcohol consumption, and in the 20–39 age group it is responsible for 13.5% of deaths [2,3]. In Poland, the results of the RAPS and RARHA screening tests indicate a declining percentage of people consuming alcohol in a risky manner—from 18.6% to 14.2% [4]. However, according to the last report published by WHO and other publications, alcohol addicts constitute 2.2% of the Polish population over 15 years of age [5,6]. Alcohol abuse and alcohol dependence constitute serious health and social problems in many countries. Drinkers are 1.5 times more likely to develop diabetes and cardiovascular diseases than non-drinkers [7,8].

Alcohol dependence equally affects the physical and mental health of an individual, disrupting their proper functioning. Alcoholism leads to the breakdown of the content of life, and by limiting the freedom of action and achievement of the goals chosen, leads to a reduction in the quality of life [9]. Most often, addicts wish to maintain good physical activity and lead an independent life as long as possible, but it is very often difficult [10].

The salutogenic concept of health developed by Antonovsky focuses on the search for resources and factors supporting health. This model assumes the integration of physical health and mental health determinants, such as coping with stress, perceiving meaning, and coping with changes. The main construct in the Antonovsky’s model is the sense of coherence (SOC) [11]. It means the global orientation of a person, expressing the degree to which the person has a permanent, but dynamic sense of understanding (perception of external and internal stimuli in a structured and coherent manner), a sense of manageability (available resources allow for an adequate response to stimuli) and a sense of meaningfulness (emerging requirements constitute challenges worth the effort and commitment). A high level of SOC guarantees maintaining balance despite experiencing difficult life situations and enables a person to motivate him/herself in the event of an illness and undertake pro-health behaviors [11,12]. The higher the level of coherence, the lower the tendency to engage in risky behaviors, including using stimulants—alcohol, tobacco products, or psychoactive substances. Individuals with high SOC levels in one study compared to individuals with a low SOC, were 28% less likely to be active smokers (OR = 0.72) [13]. Individuals with a high SOC also show greater physical activity [14,15]. According to studies, people addicted to psychoactive substances, including alcohol, show a low SOC [16,17]. In another study among patients with coronary heart disease, a low SOC before commencing treatment translated into a poorer health outcome [18].

Antonovsky defined recovery as a constructive process where the patient approaches their situation in an adaptive and future-focused manner. This definition emphasizes the essence of one’s own resources, including a high SOC, in the process of treating mental disorders such as alcohol dependence. The treatment process based on the SOC assumes the strengthening of positive life attitudes [19]. Getting out of addiction is a long-term process that requires re-evaluation of life attitudes, it cannot focus only on the aspect of physiological addiction, which is part of the concept of salutogenesis and the SOC [20].

It should be emphasized that a strong SOC is not the only determinant of success in the treatment of addicts, but it has been shown that the therapy enhancing the SOC brings positive results in the treatment and maintenance of longer abstinence by patients [21]. The salutogenic concept of health proposed by Antonovsky creates the possibility of shaping a strong SOC, which affects the length and quality of life of addicts [22]. Medical sciences should therefore become more interested in the solutions proposed by Antonovsky, not only in the theoretical context, but they should incorporate them into therapeutic practice through their activities. The objective of this study was to determine the correlation between individual factors and the SOC as well as the influence of the SOC concept on pro-health behavior in individuals addicted to alcohol. The results can be used in further work on treatment processes for people struggling with addiction, based on salutogenic talk therapy [12]. This method of therapy can be helpful in increasing coping in the recovery process among people with addictions, which relate not only to the sphere of physical but also mental health [23].

## 2. Materials and Methods

The study group consisted of 110 men aged 17 to 60 who stayed at the Department of Alcohol Addiction Therapy at Kępiński’s Specialist Psychiatric Institution of Healthcare in Jarosław. The basis for the stay in the ward in all patients was diagnosed alcohol dependence (ICD-10: F10); all but two patients agreed with the diagnosis made by the specialists and did not deny it.

Two tools were used in the study:

(a) Aaron Antonovsky’s “SOC-29 Life Orientation Questionnaire” used to assess the SOC. It consists of 29 questions grouped into three scales. It measures the general level of the SOC and its component factors, i.e., the sense of understanding, the sense of manageability and the sense of meaningfulness.
(b) Zygfryd Juczyński’s “Health Behaviour Inventory” (HBI) was used to evaluate pro-health behaviors undertaken by patients. HBI consists of 24 statements relating to various types of positive pro-health behaviors. The higher the score, the greater the intensity of the declared pro-health behaviors. The inventory includes four subscales examining proper eating habits, preventive behavior, and positive mental attitude. Internal compliance of HBI established on the basis of Cronbach’s alpha is 0.85 for the entire inventory [24].

## 3. Results

### 3.1. Study Group

110 men aged 17–65 (M = 42.53; SD = 9.50) participated in the study. Table 1 presents the characteristics of the study group in terms of sociodemographic and medical variables. Some terms in the table come from the Polish law. ‘Blue card’ is a procedure developed by the Polish police that covers all official activities undertaken and carried out in relation to a justified suspicion of domestic violence, including consequences of alcohol consumption. ‘Order to leave their place of residence’ is order from art. 275a of Polish criminal-law code. A person committing domestic violence, under the provisions of the law and the victim’s legal request, may receive a court order to leave their place of residence to ensure the victim’s safety for the duration of legal proceedings.

The largest number of respondents were married men (43.6%) and had vocational education (48.2%). Most of the respondents smoked cigarettes (86.4%). Smokers smoked from 10 to 45 cigarettes a day (M = 20.55; SD = 6.30). The number of hospitalizations of the respondents ranged from 0 to 15 (M = 2.39; SD = 3.18).

### 3.2. Descriptive Statistics

Table 2 presents descriptive statistics for the analyzed interval variables, i.e., mean values, standard deviations, minimum and maximum values, and the values of the Kolmogorov–Smirnov test, which was used to verify the assumption of the normal distribution of the analyzed variables.

There were no statistically significant differences between the analyzed variables and the shape of the normal distribution (the value of the K-S index for both variables was 0.08).

According to Antonovsky [11], scores of SOC in the range 51–100 indicate low SOC, scores in the range 101–152 indicate average sense of coherence, and scores higher than 152 indicate high SOC. The mean value in the current sample falls in the range of average scores, while minimum value falls in the range of low scores, and maximum value falls in the range of high scores. The highest possible value in the questionnaire is equal to 203. The highest value in the current sample was equal to 166.

The maximum of value in the HBI questionnaire is equal to 120 and the maximum value in the current sample was also equal to 120. The mean value of pro-health behavior in the sample from general population in Poland is equal to 81.82 [24]. According to the value of one-sample t-test the difference between the mean value of pro-health behavior in the current sample and the mean value acquired from the sample from general population was statistically significant, t(109) = −8.47, *p* < 0.001. The level of pro-health com-parison in the current sample was significantly lower.

### 3.3. SOC and the Intensification of Pro-Health Behaviours

The respondents were divided according to the age median, i.e., 42, into two groups: 17–42 years old and 43–65 years old. Table 3 shows the Pearson’s r correlation coefficients between the SOC and the intensity of pro-health behaviors for selected sociodemographic variables: age (in the group of younger and older individuals), education (in the group of individuals with primary or vocational education and in the group of individuals with secondary and tertiary education), place of residence (in the group of individuals living in towns/cities and villages) and in total in the entire study group.

On the basis of the obtained results, it was found that the SOC of the respondents correlated positively with the intensity of pro-health behaviors. This correlation was found for the following variables: age—in the group of older individuals, i.e., 43–65 (*p* = 0.030), education—in the group of individuals with primary or vocational education (*p* = 0.015), and place of residence—in the group of individuals living in villages (*p* = 0.028).

However, no statistically significant correlation was found between the SOC and the intensity of pro-health behaviors in the group of younger individuals (17–42), in the group of individuals with secondary or tertiary education, or in the group of individuals living in towns/cities.

**Figure 1:**
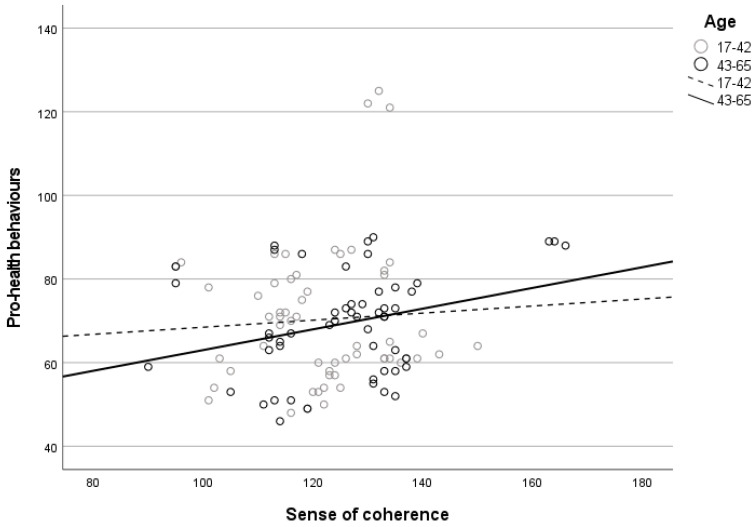

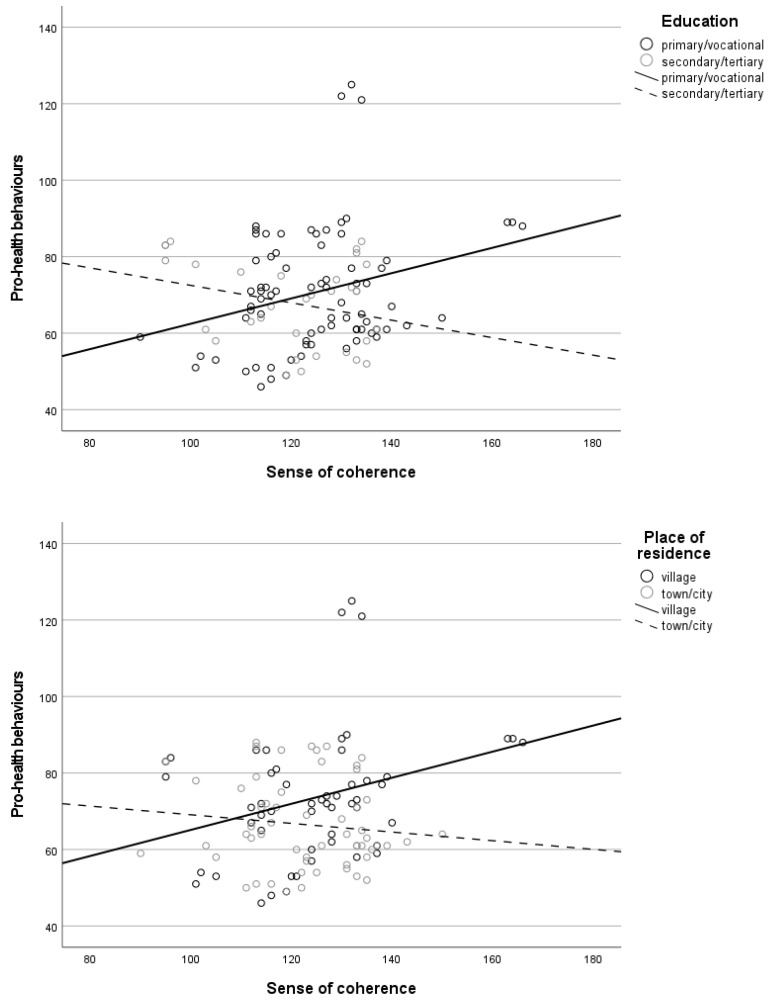
The correlation between SOC and the intensity of pro-health behaviors depending on the age (1), education (2), and place of residence (3) of the respondents.

The greater the SOC in the group of older individuals, in the group of individuals with primary or vocational education, and in the group of individuals living in rural areas, the greater the intensity of pro-health behaviors (see Figure 1).

## 4. Discussion

Alcohol dependence has a negative impact on many areas of human life, and it also has social and economic consequences. The most important psychosocial effects of alcohol dependence include problems in the sphere of family and interpersonal relationships, disturbances in economic and professional functioning, and breaking legal norms. For this reason, addictions constitute a special type of health problem, affecting many spheres of the addict’s functioning, not only in the physiological aspect [25].

According to the literature, a high level of the SOC strengthens pro-health activity and plays an important role in the management of resources needed for the implementation of intentional health-related behaviors [20].

A high level of the global SOC is associated with pro-health behaviors, and a lower level with a tendency to engage in anti-health behaviors [12]. A high SOC is associated with a tendency to avoid risky behaviors related to alcohol consumption and the use of psychoactive substances. Results of one study shows that in individuals addicted to psychoactive substances, a lower level of SOC was noticeable. The SOC was strongly associated with a reduced probability of risky alcohol consumption in both men and women [16]. In the results presented in one study, a low SOC was associated with lifestyle factors such as the amount of alcohol consumed, smoking, and lack of physical activity, which translated into a higher risk of death [26]. In the results obtained in one article, individuals with a high level of the SOC were less exposed to risk factors, led a healthier lifestyle, smoked less, were more physically active, and consumed more healthy foods [27].

In our study, the SOC was greater in the older age group than in the younger age group (r = 0.299; *p* = 0.030), which is consistent with the results obtained in two cross-sectional studies [28,29]. It is known that the level of the SOC changes throughout life, however, a higher level of coherence is observed in the elderly [18]. These results question Antonovsky’s thesis that the SOC develops until the age of 30 and then remains at a constant level [20].

The impact of the level of education on the SOC in this study indicates a negative correlation. Individuals with primary/vocational education showed a greater SOC (r = 0.276; *p* = 0.015) than individuals with secondary or tertiary education. In the part of one study where the sociodemographic variables were analyzed, a greater SOC was observed in individuals who had a university degree or no correlation was identified [13,18,20,29].

The results of this study show a correlation between the place of residence and the SOC in the case of individuals from rural areas (r = 0.303; *p* = 0.028). There are only a few studies available in the literature verifying the correlation between the place of residence and the SOC. The results obtained in one study indicate significant differences in the level of the SOC between individuals living in cities/towns and rural areas. It was influenced by several factors other than sociodemographic variables, such as the internal resources of an individual (optimism, self-esteem, sense of humor), social support, and social activity. Depending on the psychological resource, the SOC was correspondingly greater for individuals living in towns/cities or villages. In the study people living in rural settings showed greater social capital and a higher rate of participation in community activities, also residents with strong attachment to their living settings were likely to have high SOC. [30]. It can be assumed that the results of our study were also influenced by other, unidentified factors here, such as social capital, social support, or individual psychological resources, thanks to which individuals living in rural areas obtained better results than individuals from towns/cities. Psychological resources, such as self-esteem, and social resources such as support network and community activities, can explain the differences [30,31]. Higher self-esteem is a strong indicator which can lead to better results in SOC [30,31]. The same psychological resources could be influencing higher sense of coherence in individuals with primary/vocational education in this study.

## 5. Limitations

This study has a few limitations. The main on is that the study group was comprised of males only; therefore, no comparison of results between genders is possible. The small number of participants also prevents more intra-group comparisons. Furthermore, there is only one point of data collecting so the comparison cannot be made to compare changes over time, but the aim of the study was not to check the effects of the patients’ treatment, but to check their level of SOC in the ongoing disease and to consider further use of this resource in addiction treatment.

## 6. Conclusions

Individuals addicted to alcohol are characterized by a low SOC and a low level of pro-health behaviors. Among individuals addicted to alcohol, the SOC is varied. Some of the variables are related to the SOC and the intensity of pro-health behaviors. Detailed knowledge of their implementation requires further detailed research in this area. The obtained results may constitute the basis for health education and can be used to prepare patients for independent life in sobriety. Building permanent structures of internal resources based on Antonovsky’s salutogenic health concept may find application in addiction therapy, especially by introducing salutogenic talk therapy. The therapy program is based on strengthening the SOC. The main goal of the therapy is to increase patients’ awareness and trust in their inner potential, build resources and use them to increase the SOC. The salutogenic approach encourages the search for inner resources that can help to effectively reduce tension in challenging situations for the patient and focus on the ability to adapt [12]. The results of this type of intervention indicate positive changes in the daily life of the people undergoing treatment. The results of the evaluation showed that 85–95% of the participants of the therapy believed that participation in salutogenic talk therapy improved their mental health [12]. Other studies have found that in addiction therapy, setting goals based on SOC can improve therapeutic outcomes [32]. SOC can be also used in psychoeducation in developing tools for coping with stress and developing the ability to recognize its symptoms [33].

## Figures and Tables

**Table 1:** Characteristics of the study group.

Variables		*n*	%
Marital status	single	39	35.5
	married	48	43.6
	divorced	23	20.9
Education	primary	24	21.8
	vocational	53	48.2
	secondary	12	10.9
	tertiary	21	19.1
Place of residence	village	53	48.2
	town/city	57	51.8
Smokers		95	86.4
Hospitalized		81	73.6
An order to leave their place of residence		51	46.4
Blue card has been issued		22	20.0

*n*—number of people; %—percentage of the group.

**Table 2:** Descriptive statistics for the analyzed interval variables.

Variables	M	SD	Min	Max	K-S	*p*
Sense of coherence	123.71	13.58	90	166	0.08	0.051
Pro-health behavior	69.89	14.76	46	120	0.08	0.064

M—mean value; SD—standard deviation; min—minimum value; max—maximum value; K-S—the value of the Kolmogorov–Smirnov test; *p*—statistical significance.

**Table 3:** Correlation coefficients between the sense of coherence and the intensity of pro-health behaviors.

Variables		*r*	*p*
Age	17–42	0.060	0.657
	43–65	0.299	0.030
Education	primary/vocational	0.276	0.015
	secondary/tertiary	−0.279	0.115
Place of residence	village	0.303	0.028
	town/city	−0.119	0.378
Total		0.158	0.100

## Data Availability

The data analyzed during the current study are available at the authors.
